# If Veganism Is Not a Choice: The Moral Psychology of Possibilities in Animal Ethics

**DOI:** 10.3390/ani10010145

**Published:** 2020-01-16

**Authors:** Silvia Panizza

**Affiliations:** School of Philosophy, University College Dublin, Belfield, Dublin, Ireland; silvia.panizza@ucd.ie; Tel.: +44-791-748-64-64

**Keywords:** moral psychology, veganism, moral possibilities, imagination, decision-making, fact and value, ought implies can

## Abstract

**Simple Summary:**

Discussions about the ethics of buying and consuming animal products normally assume that there are two choices equally available to moral agents: to engage or not to engage in such behaviour. This paper suggests that, in some cases, the experience of those who refuse to participate in animal exploitation is not a choice, but a reconfiguration of their understanding of what animals, and the products made out of them, are. Such reconfiguration involves not seeing animals as something to eat, wear, control, etc. Hence, it is not always correct to speak of veganism as a choice: the reason being that, sometimes, the opposite does not present itself as a possibility.

**Abstract:**

In their daily practices, many ethical vegans choose what to eat, wear, and buy among a range that is limited to the exclusion of animal products. Rather than considering and then rejecting the idea of using such products, doing so often does not occur to them as a possibility at all. In other cases, when confronted with the possibility of consuming animal products, vegans have claimed to reject it by saying that it would be impossible for them to do so. I refer to this phenomenon as ‘moral impossibility’. An analysis of moral impossibility in animal ethics shows that it arises when one’s conception of ‘what animals are’ shifts—say through encounter with other animals. It also arises when individuals learn more about animals and what happens to them in production facilities. This establishes a link between increased knowledge, understanding, and imaginative exploration on the one hand, and the exclusion of the possibility of using animals as resources on the other. Taking moral impossibility in veganism seriously has two important consequences: one is that the debate around veganism needs to shift from choice and decision, to a prior analysis of concepts and moral framing; the other is that moral psychology is no longer seen as empirical psychology plus ethical analysis, but the contents of psychological findings are understood as being influenced and framed by moral reflection.

## 1. Introduction

As the field of animal ethics has been expanding, so has that of moral psychology. The two areas of ethics, however, have not frequently met, as lamented in a recent book by T.J. Kasperbauer [1], who aims to bridge that gap. We need to know more about what people are actually like, the book’s contention goes, in order to make sure that the moral theories we adhere to in relation to animals and the requirements and prohibitions they command are realistic, i.e., suitably constrained by the facts that psychology, independently, provides us with.

My approach … aims to take psychological constraints seriously when formulating moral prescriptions. Whatever one’s moral goals, they have to be constrained by our moral psychological profiles, by the actual processes employed when we judge what is right and wrong.

[1] (pp. 10–11, emphasis added)

To this end, Kasperbauer suggests that animal ethicists should follow the ‘ought implies can’ (OIC) model, stated thus: “I have a certain moral obligation (like refraining from eating animals), only if I can actually meet that obligation” [1] (p. 114). In order to clarify these constraints, Kasperbauer turns to the empirical findings of moral psychology, such as studies of individual and group behavior and moral motivation. The conclusion is that animal ethics has been overly optimistic about people’s ability to either care for animals or act in favour of animals in the way that most animal ethics theories recommend. In particular, theories that commend an ‘ideal’ should be abandoned, because there is no way for the ideal (by definition, and empirically) to be met. Even demands that are ‘in principle’ possible, but unlikely to be met, are unlikely to satisfy the OIC principle, and should not be made by ethics. These demands, therefore, represent, in this view, a serious flaw of the theories, so new ones should be developed, or the old one adjusted, to make demands that are possible to meet. 

The aim of this paper is to turn all these claims, and the philosophical assumptions on which they lie, on their heads, through an examination of the, to date, little discussed important moral-psychological phenomenon of ‘moral impossibility’. Taking moral impossibility seriously, I will argue, it suggests a radically different way of thinking about a specific and increasingly adopted position in animal ethics—not contributing to animal exploitation by buying or consuming animal products, which I shall call veganism for short—which entirely subverts the view just summarized. The outcome is a dual challenge to mainstream animal ethics, on the one hand, by making room for a different understanding of fact and value and of the relation between ethics and psychology; on the other, by offering an account of a specific phenomenon in veganism and the moral psychological limits in our moral relationships with animals. The benefits of this reversal are both that it allows us to better account for the experience of individuals who have rejected animal exploitation for some time, and that, as a consequence, it offers a new way to approach the debate (increasingly polarising, particularly, in its public form) between supporters and opposers of veganism. 

(Two clarifications about the concept of veganism are in order: first, the definition offered here is in accordance with that of The Vegan Society, but it is to be taken as a working definition, acknowledging that veganism can be both broader than that (extending to any action which harms animals with many degrees of indirectness) or narrower (allowing the consumption of some animal products, depending on how narrowly ‘exploitation’ is defined); however, these are discussions to be developed elsewhere. Second, although ethical veganism (and vegetarianism) is sometimes motivated by other concerns, this paper focuses on veganism based on moral concern for non-human animals, which appears to be the most cited reason for veganism: see e.g., Ruby [2].) 

The key concepts under analysis are ‘possibility and ‘impossibility’, while the grounding experience that focuses the discussion is that of moral impossibility in consuming animal products on the part of vegans. The reported experience of impossibility in this particular case is presented as closely linked with moral change: on the one hand, it is experienced as a form of moral transformation; on the other, it tends to occur as a change of habit, rather than a continuation of old forms of behaviour. I will argue that close attention to the role of impossibility in animal use leads to a reconfiguration of the debate on veganism by moving it away from the standard model of opposing choices and deliberative conclusions based on conflicting reasons (employed by utilitarian and deontological theories alike), towards a prior and more fundamental model based on individual framings of possibilities, which are, in turn, grounded on different moral understandings of what non-human animals are. The conflict of ethical positions and conclusions, in other words, cannot be properly understood without an investigation of what frames the possibilities that each position takes into account. 

## 2. The Priority of (Psychological) Fact over Value

According to a widely-held assumption in ethics, expressed in the context of animal ethics by Kasperbauer, one of the key contributions that the empirical study of moral psychology can make is that it ‘constrains’ what ethics can demand by offering an understanding of what it is plausible that people will do. This is so according to the often cited ‘ought implies can’ principle. Kasperbauer rightly suggests that the scope of ‘can’ is not clearly settled by the principle. His suggestion is that, besides strict impossibility, we should also exclude from the demands of morality those actions that are psychologically very unlikely, such as extending empathy to all animals or ending all forms of animal exploitation—the latter represented by the ‘abolitionist approach’, championed by Francione and Charlton [3], and Steiner [4]. Moral impossibility has, in this model, the following features:

1. It is discoverable empirically, prior to moral thought.
2. It provides constraints to ethics (where ethics is considered as primarily consisting of obligations).
3. It extends beyond what is strictly impossible to what is, currently, rather difficult to achieve for the groups studied. (The level of difficulty is not clearly specified).

All of these points should be challenged. The meta-ethical framework underlying this model is a strict fact and value distinction, where possibilities, including psychological ones, are determined empirically and independently of moral thought. Ethics is limited to the role of offering obligations or prohibitions by observing, from the outside in, the world of facts described by the empirical sciences—with psychology among them. This view of facts and values is the underpinning of the mainstream moral theories in the West, being shared by both deontology and consequentialism. This model is also assumed, without blinking, by Kasperbauer, who writes that “of course it is true that descriptive facts do not directly translate into normative prescriptions” [1] (p. 8).

However, this model does not seem so obvious to everyone. It was challenged, for instance, by Iris Murdoch over seventy years ago, who called it, at the time, the ‘current view’, in response to R.M. Hare’s prescriptivism, and the anti-realist whole-world view it represented:

In this view, the moral life of the individual is a series of overt choices which take place in a series of specifiable situations. Further, a moral judgment is one which is supported by reasons held by the agent to be valid for all others placed as he, and which would involve objective specification of the facts available to disinterested scrutiny.

[5] (p. 34)

Murdoch responds that such a view of morality was just one possible view, and that it was motivated not so much by inevitable conclusions drawn from observation and reflection, but from the desire to think of ourselves as ‘free’ agents, in a world dominated by liberal values, which are well accommodated by this view. By contrast, she argues that much of what we ordinarily think of as the *locus* of morality is obscured by that model. Two important items, in particular, are missing: the moral relevance of the individual person’s general ‘mode of being’, and the fact that our very way of conceptualizing what the facts are, and indeed of choosing which facts to reflect on as available options, is the result of a moral process:

When we attend to the notion of ‘moral being’ as self-reflection or complex attitudes to life…moral differences look less like differences of choice, given the same facts, and more like differences of vision. … like a total difference of Gestalt. We differ not only because we select different objects out of the same world, but because we see different world.

[5] (pp. 40–41)

These contrasting models have significant consequences for how we understand what is possible or impossible for us, morally thinking beings, when it comes to morally relevant possibilities. One such morally relevant possibility is the consumption of animal flesh and other animal products.

## 3. Moral Impossibility and Eating Animals

There is an important experience in the domain of ethically informed human ways of relating to other animals, which the orthodox model of moral psychology, and its application of the OIC model, are unable to explain. This is the case of moral impossibility, where the impossibility is not one that is morally neutral and works to constrain moral demands, but one that is itself *determined* by moral demands. I suggest that, in veganism, moral impossibility emerges in two ways: (i) as the exclusion of certain possibilities from the range of available options; (ii) and as the inability to consider a course of action as a real, morally available, option. In the first case, certain possibilities (e.g., consuming animals) simply do not occur as possibilities to some people, not because of physical or psychological constraints, but because of moral commitments, reflections, intuitions or general way of being. In the second case, the previously absent possibility, such as that of consuming animals, presents itself from the outside: one sees what others do. In that form, the moral impossibility takes on a first-personal valence: it is accepted to be possible for others, but not for oneself, to engage in a certain type of behaviour. (This is discussed in relation to the concept of the ‘unthinkable’ in Section 5 below). This second case, where one recoils from seeing something as possible for others, may lead to the first case, where one does not ordinarily conceive of that action among one’s possibilities, or vice versa, so the boundaries between the two cases may blur. Moreover, in both cases, purchasing or consuming animal products is impossible in this sense: for (at least some) vegans, it does not present itself as an option.

While the phrase ‘moral impossibility’ may seem strange, what it describes, in at least the first sense just explained, is a perfectly ordinary phenomenon: our range of options is always narrower than the theoretically, physically possible range of options, and moral framing is one of the factors that narrows down the range. Just as we do not consider trying out a new knife by stabbing the neighbour, but choose, say, among the food items in our cupboard, so for some people the choice of what to have for dinner does not involve the consideration of animal products at all.

I would go as far as claiming that some form of moral impossibility, as I construe it, is always part of morality. However, minimally, we can say that, in some situations, especially when something important is at stake, impossibility is a real phenomenon, and that in some of those situations, such phenomenon can be both epistemically and morally beneficial.

In the domain of animal ethics, the language of impossibility is invoked by those who define themselves as ethical vegans, to describe their stance in relation to animal exploitation or in explaining how they originally switched from more conventional habits which include the use of animals, to a refusal of those habits. Here is an example from a study of vegetarians and vegans, who were asked if they would consider eating meat in social situations to avoid embarrassment. Not only did most participants said they would not, but some claimed that they *could* not:

When asked whether they would eat or refuse a meal containing meat in social circumstances where it was very difficult and embarrassing to refuse … most respondents said they would not eat the food … even if this would cause considerable embarrassment. … For some it was virtually impossible for them to eat the meal:

I just find the idea of eating a dead animal just horrible. Even to be polite socially I just couldn’t do it. If I went round to somebody’s for dinner and they didn’t know I was vegetarian … I couldn’t eat a sausage or a steak. I just couldn’t. I would have to say look I’m sorry but I just can’t eat this (SB1).

[6] (pp. 161–163)

And here is what Michael Pollan, describing a visit to a feedlot, writes:

Standing there in the pen alongside my steer, I couldn’t imagine ever wanting to eat the flesh of one of these protein machines. Hungry was the last thing I felt.

[7] (p. 84)

We still need to properly understand the meaning and force of this ‘cannot’, which requires, first, distinguishing it from other kinds of impossibilities, and secondly, explaining its specific ethical character in a way that is not simply an exclusion of perfectly reasonable options. 

## 4. Does Ought Imply Can?

Moral impossibilities are distinguished from both moral choices and strict impossibilities for a number of reasons:

1. First, they are indeed impossibilities, rather than choices, made by us or by others for us, regarding what is not desirable. This is so both phenomenologically and in terms of the ability to predict the actions of the subject: as Bernard Williams [8] points out, moral impossibilities are not mere predictions about oneself, but they make such predictions possible in a way that moral choices do not. (Williams is here discussing the phenomenon of moral incapacity, which coincides with the second form of moral impossibility identified above: the case in which one can see that something is, in principle, possible, but cannot bring oneself to do it. Williams explains this type of impossibility through the role of deliberation: moral incapacities are incapacities one does or would, on reflection, endorse; they fit with one’s conception of the good.)
2. Second, unlike strict impossibilities, they are made impossible neither by logic nor by the laws of nature (e.g., in the latter case, living to be 500 years old would be impossible for a human being).
3. Thirdly, these impossibilities are not grounded on empirical, impersonally available, external facts, which, if removed, would also remove the associated impediment.

If we examine the language of the two examples in the section above, we discover two further relevant features of moral impossibility:

4. In the first example, impossibility is not presented as a result of deliberation, but as blocking another possible reason, i.e., that of pleasing the dinner host and avoiding social awkwardness. We do not have one reason trumping another, but impossibility simply removes other reasons from consideration.
5. In the second quote, Pollan is talking not only about being unable to eat one of those animals, but not being able to *imagine* eating them. It is not only his actions, but his imagination, which is blocked here.

I shall return to the last two points later. First, let us look at the second and third ones, in relation to how they distance this kind of moral impossibility from the merely psychological one offered by Kasperbauer, and the consequences of the distinction.

Kasperbauer invokes psychological impossibility as part of his argument in support of the OIC principle. The aim is to show that, if something is psychologically impossible, based on his view of impossibility, then it should not be morally required. One way to put this claim into question is to challenge the OIC principle. As Kasperbauer acknowledges, the principle already has its critics. Flanagan [9], for example, argues against the OIC by noting the temporal relevance of impossibilities. Even if it is psychologically true that something is impossible now, it does not mean it will be psychologically impossible in the future. Gilabert and Lawford-Smith [10] also take into account the importance of future developments, discussing the ‘feasibility’ of pursuing particular outcomes over longer periods of time. An ‘ought’, then, can refer to future behaviour. Sinnott-Armstrong [11] goes further and rejects the constraints of the ‘can’. It may still be the case, he claims, that we have a moral responsibility or a duty even if we are unable to meet them, for ‘ought’ is not conceptually implied by ‘can’. From a different angle, Brownlee [12] takes the discussion into the space of ideals, relieving the pressure of feasibility from ideals, which are understood as guiding ideas, rather than specific states of affairs to be achieved: their role is to inspire and to change the moral agent. 

I want to present a different challenge to the OIC, particularly as it occurs in animal ethics. Rather than claiming that it is possible to have an obligation even if it cannot be achieved, the discussion of moral impossibility aims to explore the idea that what it means to be able to do something can be thought about differently than the OIC framework allows. Specifically, that we may be too hasty in accepting possibility and impossibility as something that can be established *before* morality comes in. 

In opposition to the critics of OIC, Kasperbauer suggests we should widen the scope of impossibilty. Besides excluding ‘strict’ types of impossibilities (primarily, in this context, physical ones), OIC needs to take into account what is only loosely called impossible, i.e., what human beings tend to find too difficult to achieve. In a critical discussion of the widespread and rather unexamined use of OIC, J.W. Smith [13] makes what appears to be precisely the same point: that the force of OIC in moral theories is not, primarily, that of strict impossibility, because in those cases (such as living to be 500, or, more to the point, fulfilling a promise to run a charity marathon when one’s legs are severely injured), there is no meaningful sense in which we could ask the question: “ought you?”. Strict impossibilities remove the context in which the OIC question can even arise in any informative sense.

However, Smith argues, for OIC to have the moral force that it does, in offering something helpful to moral reflection, the ‘can’ it contains has to refer to something which it is reasonable to expect of someone, and that, he concludes, involves in itself a moral judgment. This analysis of OIC shows that the model which takes for granted that we can identify possibilities impersonally and amorally, on which the OIC appears at first sight to be grounded and endorsed by Kasperbauer, actually denies any real force to the very rule it tries to uphold. Rather than limiting any ‘ought’ to a ‘can’, the idea of OIC is that, as Smith puts it, “the most important ‘cannots’ of all conceal further ‘oughts’” [13] (p. 375): “The whole force of the ‘cannot’ is moral” [13] (p. 372).

The kinds of moral impossibilities I am considering, those relating to the consumption of animals, clearly illustrate how, beyond strict (and rather uninteresting, at least morally) impossibility, what is possible cannot always be established outside of moral considerations. Indeed, these cases display an opposite movement, not from can to ought, but from the experience of impossibility, to the articulation of a different way of thinking about moral positions and moral discourse in general. Let us see how.

## 5. The Unthinkable

The first observation of impossibility in the case of vegans was the idea that, when it comes to choosing what to eat (or wear, or buy, etc.), the possibility of eating or buying animal products does not occur to some of these individuals. It is not part of the world as they perceive it at that particular moment. Since that is not strictly impossible, but also not a moral choice, which would require considering and then discarding those optionss, what is this phenomenon? We can seek an answer by returning to Iris Murdoch, who offers a reflection on this question in *The Sovereignty of Good*. She writes:

I can only choose within the world I can see, in the moral sense of ‘see’ which implies that clear vision is a result of moral imagination and moral effort.

[14] (p. 37)

This statement is made in opposition to precisely the view, which is represented by the standard theories in animal ethics and supported by the use of OIC I am questioning, that when deliberation occurs, all the facts are available to the moral agent. Choice emerges here as the activity of a thin will, unbound by facts, which surveys the world from above, detached from all human concerns, and then picks out one of the empirically available options, based on reasoning and deliberation [14] (pp. 1–9). That is not, Murdoch observes, how we normally live our lives, our moral lives, where morality is a frequent, if not constant, presence, not only in the form of rules and choices, but more fundamentally—to use a Murdoch-typical expression—in the very ‘fabric of our being’. 

Murdoch’s claim in the quote above points to a fundamental structuring and shifting, opening up and narrowing down, of possibilities, which is not a result of choice, but on the contrary, takes place *before* any choice appears available to us. The moral impossibility that is part of veganism, I argue, takes place in both a narrowing down and an opening up of possibilities, and in fact in the former via the latter (see Section 7 below). 

As we saw, moral impossibility includes both the fact that certain possibilities do not present themselves at all, and the fact that even when they do, they do not appear as real possibilities to the subject. In the second case, the subject is aware that something is being done, but fails to understand how anyone can allow that action into the space of possibilities. The action is not rejected as wrong, but considered ‘beyond the pale’, ‘mad’, ‘unthinkable’: this is how both Raimond Gaita [15] and Harry Frankfurt [16] characterize those actions that, for their extreme nature, fail to make sense within someone’s ordinary moral framework. My point is that, for many vegans, the consumption of animal products takes on precisely this dimension. The experience of impossibility is more likely to occur after some time from the moment of becoming vegan, which may itself be a gradual shift. The argument allows that, at some point, an overt, ordinary choice may have occurred. What matters is that, at a certain point in time, the individual comes to have a different moral outlook, which is expressed, crucially, by the actions she takes as possibilities and those she does not. As Williams put it, impossibilities are not just outcomes, but *expressions* of the moral life [8] (p. 59). 

The ‘unthinkable’ figures in the title of the recent BUAV/Oxford Centre for Animal Ethics report on scientific experiments on animals, ‘Normalising the Unthinkable’ (2015):

11.1. The deliberate and routine abuse of innocent, sentient animals involving harm, pain, suffering, stressful confinement, manipulation, trade, and death should be unthinkable. Yet animal experimentation is just that: the ‘normalisation of the unthinkable.’

11.11 This normalising of the unthinkable needs to be de-normalised and de-institutionalised.

[17] (pp. 70–72)

The title comes from an article by Peattie from 1984 [18], in which she argued that the planning for possible nuclear war should not be a possibility to be considered, and yet that society’s calm and collaborative attitude created an atmosphere in which what ought to be beyond the range of morally considerable options appeared normal. The idea that the unthinkable can be normalized shows clearly that it is possible, factually possible, for animal exploitation and harm to happen. In fact, it does happen. It is also empirically possible to engage, again, in practices contributing to animal suffering. In fact, those who reject them now probably engaged in them in the past. From an impersonal, bird’s eye perspective, all that is possible, just as it would have been possible for Luther to recant. (‘Here I stand, I can do no other’ is often taken as the motto for debates of practical necessity, the other side of the impossibility I am discussing here. See the volume *Dimensions of Practical Necessity: “Here I Stand. I Can Do No Other”* [19].) However, he could not. And when it comes to getting involved in animal pain and death, sometimes, some people cannot. However, still, why ‘I cannot’, instead ‘I won’t’, or ‘I ought not’? As Williams [8] observes, it is not simply a matter of intensifying the moral prohibition, it is not a matter of ‘I really, really don’t want to, because it’s really, really wrong.’ We need to look further. 

## 6. How the Moral Imagination Determines Possibilities

This is where Murdoch helps us. The idea that we can only choose within the world we can see can appear so commonsensical as to seem banal. Of course I cannot choose something that I am not aware of. Nor will I choose something that I do not consider as a reasonable option. (To use Murdoch’s metaphor, if morality is like a visit to a shop, where we survey the options and then choose, we will not buy pears if they are hidden behind the counter; nor will we buy the furniture, which is visible but not something that is for sale—cf. [14] (p. 8). However, Murdoch adds: “in the moral sense of see.” Which involves, in her words, “moral imagination” and “moral effort.” (This explains why morality is precisely *not* like a visit to a shop: the stock, if you will allow me to push the metaphor, is not selected by someone else). 

Our imagination can frame the world and deepen our understanding in various ways, and in doing so, can either broaden or restrict our options. This happens all the time: for instance, when we decide what to do in our spare time, we may consider going for a walk, or watching a film, or visiting a friend. However, there are things we do not consider. What we do not consider, among other things, is burning cars parked in the street, or making fun of homeless people. The fact that we do not consider those is not normally regarded as blindness or lack of imagination on our part. It is also not normally regarded as a choice. Similarly, for a vegan, cooking a piece of veal or going to a bullfighting show are simply not options. It is not that they are considered and then discarded. They never present themselves to them as possibilities at all. 

Another way of putting this, as highlighted in the quote from Pollan, is that those people’s *imagination* does not include those possibilities, it does not take them there (see point 5 above, in Section 4). Moral imagination, presented by Murdoch as one of the components of clear vision, is the faculty that allows us to ‘grasp’ the world through conceptual framing, which can be more or less appropriate. In this sense, the imagination is a basic faculty of perception and understanding, and it is moral insofar as, Murdoch suggests, moral concerns pervade most of our encounters with the world. (To the extent that use of the imagination is unavoidable in understanding, Murdoch comes close to Kant, but immediately departs from him in taking the activity of the imagination to be inherently moral [20] (pp. 308–316).) However, in framing reality and making it available to the understanding, the imagination also identifies objects and situations in the world as containing possibilities, and identifies some possibilities while excluding others. As Murdoch puts it, the imagination is “a type of reflection on people, events … which builds detail, adds colour, conjures up *possibilities* in ways which go beyond what could be said to be strictly factual” which is also “a sort of personal exploring” [21] (p. 48). 

Possibilities are determined by individuals’ moral imagination, which frames the world in particular ways, and can be more or less truthful. However, how does the moral imagination work in the case I am discussing, eating animals and using them? How do we know that not taking that as an option is not, as I mentioned, a lack of imagination, a form blindness? This question will take us to consider the claim made at the start, that moral impossibility can represent, in some cases, both epistemic and moral progress. 

## 7. Impossibility and Knowledge

Earlier I talked of a narrowing down and an opening up of possibilities, and claimed that these two ‘movements’ are linked, and that narrowing down may be a result of increased understanding and inclusion of other sorts of possibilities. On Murdoch’s view of the moral imagination, unlike the impossibility derived from blindness, imagination deepens our sight in some ways, while removing other things from our field of vision. Or it does the latter through the former. It is this, I am arguing, that happens when one stops using animals as resources.

I have offered a partly descriptive account of moral impossibility, following what I argue may be better described as a moral transformation rather than a moral choice. This account seems to prevail in the context of our relation to other animals and in other cases where the moral issue is of a fundamental or extremely important nature. To describe the phenomenon as a ‘transformation’, inheriting the Platonic language of *periagoge*, or ‘turning around’, is to imply two elements: one is that the turning around, in the Platonic sense, is broadly ethical; the other is that the transformation is leading closer to, rather than away from, truth and virtue. For this to be justified, it is necessary to see how impossibility involves, parallel to the narrowing down of other options, a broadening of our field of vision, particularly through a deepening of our imaginative faculties. I will start from the second point. Let us go back to Murdoch’s quote again:

I can only choose within the world I can see, in the moral sense of ‘see’, which implies that clear vision is a result of moral imagination and moral effort.

[14] (pp. 36–37)

Let us now pick out two other phrases: ‘clear vision’ and ‘moral effort’. What Murdoch is claiming is that the limits, as well as the shape of our world, are defined by a moral imagination which we are partly responsible for, which partly defines us, and which aims, and is able to achieve, progressively greater clarity and truthfulness. Those who reported a change of mind, or heart, or a change of habits, in relation to animal use and consumption, in the overwhelming majority of cases, did so after exposure to information about either (a) other animals’ lives, characteristics and behaviour, or (b) the treatment and conditions of other animals used for human purposes in farms, labs, etc.; or both.

As recent studies led by Amelia Cornish [22] have shown, the average knowledge of the animal industry among consumers in developed countries is, generally, very poor. At the same time, a Eurobarometer research [23] has found that the correlation between such knowledge and the rejection of animal products is very high. These studies establish a direct link between what we know about animals and their situation, and the refusal to engage in behaviour that contributes to their exploitation.

Abstract knowledge, however, is not always sufficient to motivate a behavioural change, and it is particularly insufficient to create the kind of impossibilities related to a world-view shift we are concerned with. The knowledge also needs to be endorsed and felt. This further link is provided by the correlation observed between concern for animal treatment and animal presence. For instance, Paul and Serpell [24] showed how living with companion animals, especially as children, increased adults’ moral concern for other animals. Phillips and McCulloch [17] discovered that while perceived sentience ratings depend on similarity to humans, research participants were more likely to rate dogs far more highly than pigs, despite pigs’ developed cognitive capacities, showing the role of familiarity in understanding what another animal is like. All of this is broadly in accord with Allport’s famous ‘contact hypothesis’ [25], according to which first personal contact with members of another group leads to better understanding, including empathetic understanding.

From this, we can derive a picture whereby presence and affective engagement lead to greater appreciation of the capacities of other living beings, capacities which can also be established empirically, but which are not generally acknowledged without the two elements just mentioned. Here, acknowledgment and understanding, as opposed to abstract knowledge and awareness, are key. So is the effort not to look away, or ignore the information one is presented with. Such effort is moral, not only because the information one finds challenging is morally relevant, but because it requires overcoming previous moral positions, which could have been held more or less overtly, and accepting the possibility that one’s previous actions were morally problematic.

From the idea that proximity can promote understanding, by adding the fact that proximity, in the case of animal consumption, is one of the central factors leading to change, and precisely what is lacking in most consumers’ experience, we can derive the conclusion that, in these cases, moral change is the result of deeper or wider understanding. This is the ‘greater accuracy’ of the moral vision Murdoch is advocating, resulting from moral imagination and moral effort.

Of course proximity does not always lead to change. As Kasperbauer points out, in relation to the ‘contact hypothesis’, contact with another group can also increase conflict if the group is perceived as hostile [1] (p. 185). However, the fact that when change is present, often so is proximity, physical or mental and emotional, is a prima facie reason to take the moral conversion of giving up animal use to be one grounded in a broadened vision. A broadening which goes, as I claimed earlier, hand in hand with the narrowing down, which is better described as both the refusal to acknowledge as possible something which many others take to be so, and the exclusion of such possibility from one’s own daily life. 

## 8. Conclusions: Reframing Debates in Animal Ethics

The main claim of this paper is that the phenomenon of moral impossibility, in the context of veganism, enables us to reach a better understanding of the moral dimension of veganism, with two important consequences. One is the re-framing of the debate surrounding the use of animals as resources for human purposes. On this view, veganism does not only, and on a day-to-day basis not primarily, involve making an overt and conscious choice to avoid animal products, where consuming them is seen as possible but rejected, but rather involves a thorough shift in perspective and worldview, in which the very concept of animal, as Cora Diamond [26] famously suggested, involves the idea of being ‘not something to eat’. 

If this is so, then we may have to consider altering the debate on animal use, which is no longer exhaustively captured by the model that takes it as a disagreement about different principles applied to certain shared, available facts. Rather, the debate needs to take into account the *process*, briefly sketched here, which takes some individuals to no longer see consuming animal products as a possibility, and the process by which others take it not only as a possibility, but as a default option (in this sense, we can say that eating animals is also ‘not a choice’). The discussion will need to carefully analyze the way in which the relevant concepts employed by both parties (starting with ‘animal’, or more precisely ‘lamb’, ‘chicken’, etc.) are formed and used, which involves, to some extent, sharing the sensibility and imagination which frames those concepts. Here, we can see the role of art, for instance of literary fiction, in making available and inhabitable a particular ‘vision’. 

The other consequence of taking moral impossibility seriously is that it offers an understanding of the interaction between psychology and ethics that differs radically from the standard approach, which takes for granted a fact-value distinction and an ‘ought implies can’ principle derived from such a distinction. Veganism is precisely one of those practices that may be too morally demanding according to Kasperbauer’s reading of OIC, framed within such standard understanding of moral psychology. Apart from the move from impossibility to difficulty as part of OIC, the claim is framed within a view of facts and values that takes for granted that we *know*, and can establish through empirical psychology, quite independently of ethics, what human beings are psychologically able to do, and what is too difficult for them, and that such clear knowledge has the only goal of establishing ‘constraints’ to ethical obligations. The role of psychology is to provide descriptive accuracy, while keeping ethics in check.

However, if what is possible for us depends on moral thinking, including moral imagination and conceptualizing, as the phenomenon of moral impossibility suggests, then psychology does not, on its own, determine the facts. Facts are not prior to moral reflection. Moral reflection shapes not only what the facts are and what they look like, but also which ones are available. The experience of rejecting animal use shows precisely this. If using animals is considered morally impossible, or unthinkable, one will not see its opposite as too demanding. One will see it as a default. I have suggested that we reverse the OIC into a ‘can implies ought’ principle: one’s moral outlook shapes the world one lives in, creates some possibilities and removes others. The bounds of human psychology, therefore, cannot be determined outside of morality. Furthermore, moral reflection, moral attention, and moral conversion are all activities that moral philosophy can encourage and shape, and they will, in turn, shape what is possible psychologically. 

The relationship between ethics and psychology is not unidirectional, and taking it that way would limit and censor both psychology and ethics quite severely, (a) by limiting the role of ethics in human life to obligations, and its scope to what we can expect people to do, as opposed to see or think or feel, and (b) by limiting the contribution of psychology to ethics to the role of providing ‘constraints’ on what ethics can do, as opposed to being an open-minded and curious exploration of what human life presents, acknowledging that each ‘fact’ can be presented, understood, used, and made to count in different ways. This will involve, among other things, carefully constructing the empirical studies so that the questions asked do not take for granted, in our case, that the only possible moral change can occur only through choices and decisions (see, e.g., Ruby [2], who writes that his study researches the ‘decision’ to be vegetarian or vegan).

I have argued that the refusal to take other living beings as resources is, for many, not a choice, because the opposite is not a choice. Even less is it a personal choice, understood as a merely subjective inclination or preference. It is living in a world where other animals are perceived as ‘fellow living beings’, which means living within the boundaries of what their (individual) lives require and how we are able to respond to them. Through the moral imagination, which can be used to evoke, behind the end product (meat, cheese, coat fur, etc.), the living being who is the subject of that process, it means attending to what the competing worldview refuses to attend to. Hence, this moral impossibility requires, at the same time, a broader understanding, and a broadening of possibilities in other areas (such as engaging with animals as other selves, as companions, with pity, with shared joy, etc.). In other words, in making some possibilities available, others, incompatible with them, become impossible. What veganism guided by moral impossibility does not do is ask the other side to follow either a different inclination or a moral imposition coming from the outside. It asks, instead, to look here, to look at *this*, and, as Murdoch put it, to look again. [14] (p. 17). As long as the debate purports to address equally available possibilities which are chosen by some and not by others, it will continue to ignore the deep aspect of veganism and of living with other animals.
